# Physicochemical and Electrochemical Characterization of Electropolymerized Polydopamine Films: Influence of the Deposition Process

**DOI:** 10.3390/nano11081964

**Published:** 2021-07-30

**Authors:** Julian Kund, Sven Daboss, Tommaso Marchesi D’Alvise, Sean Harvey, Christopher V. Synatschke, Tanja Weil, Christine Kranz

**Affiliations:** 1 Institute of Analytical and Bioanalytical Chemistry, Ulm University, Albert-Einstein-Allee 11, 89091 Ulm, Germany; julian.kund@uni-ulm.de (J.K.); sven.daboss@uni-ulm.de (S.D.); 2 Max Planck Institute for Polymer Research, Ackermannweg 10, 55128 Mainz, Germany; marchesi@mpip-mainz.mpg.de (T.M.D.); sdharv@gmail.com (S.H.); synatschke@mpip-mainz.mpg.de (C.V.S.); weil@mpip-mainz.mpg.de (T.W.)

**Keywords:** polydopamine, electrodeposition, electron transfer kinetics, redox states, adhesion forces, pH dependence, Young’s modulus

## Abstract

Polydopamine (PDA) is a synthetic eumelanin polymer which is, to date, mostly obtained by dip coating processes. In this contribution, we evaluate the physical and electrochemical properties of electrochemically deposited PDA films obtained by cyclic voltammetry or pulsed deposition. The obtained PDA thin films are investigated with respect to their electrochemical properties, i.e., electron transfer (ET) kinetics and charge transfer resistance using scanning electrochemical microscopy and electrochemical impedance spectroscopy, and their nanomechanical properties, i.e., Young’s modulus and adhesion forces at varying experimental conditions, such as applied potential or pH value of the medium using atomic force microscopy. In particular, the ET behavior at different pH values has not to date been investigated in detail for electrodeposited PDA thin films, which is of particular interest for a multitude of applications. Adhesion forces strongly depend on applied potential and surrounding pH value. Moreover, force spectroscopic measurements reveal a significantly higher percentage of polymeric character compared to films obtained by dip coating. Additionally, distinct differences between the two depositions methods are observed, which indicate that the pulse deposition process leads to denser, more cross-linked films.

## 1. Introduction

Polydopamine (PDA) and PDA containing hybrid nanomaterials are used in a broad field of applications, ranging from trace analysis [1,2] to biomedical research [3], for drug release [4], and as functional material for filtration coatings [5]. The chemical and physical properties of PDA are mainly related to its plethora of functional groups such as catechol, amine, and imine groups, which are also responsible for tuning the properties of PDA by parameters such as pH [6] or applied potential [7]. To date, PDA films are predominantly formed via simple dip coating routines, immersing the substrate into a slightly basic aqueous dopamine solution (>pH 7.4), as initially presented by Messersmith and coworkers [8]. PDA is formed by the oxidation and subsequent self-polymerization in the presence of oxidizing species, whereby film thickness and properties depend on the experimental condition [9]. PDA is most likely composed of several components that are formed via the oxidation product 5,6-dihydroxyindole, as recently reviewed by Liebscher [10], or physical self-assembly pathways, as proposed by Hong et al. [11]. Messersmith’s group recently showed the polymeric nature of PDA via force spectroscopy measurements with contour lengths of the polymer chains of about 200 nm [12].

Alternatively, electrochemically induced polymerization is an attractive strategy to deposit thin PDA films at conductive substrates. Predominantely, cyclic voltammetry (CV) in oxygen-free basic dopamine solution is employed to deposit PDA [13]. Other electrochemical methods, such as galvanostatic deposition [14] and pulsed deposition [15,16,17], have been described in the literature. Although electrochemical PDA deposition is limited to (semi)conductive substrates, the electrochemical route allows fast deposition times in the range of seconds, in contrast to dip coating, which usually takes several hours to form PDA. In addition, a more homogenous morphology of the deposited films is obtained with less particulate material, facilitating excellent control of the film thickness [13]. The oxidation of dopamine at different electrode materials under different experimental conditions, such as varying pH values, have been studied [18,19,20,21]. The first oxidation steps of dopamine follow an ECE (electron–proton–electron transfer) mechanism even in strong acidic conditions [21]. Li et al., performed electrochemical quartz microbalance studies and proposed an ECECEE mechanism for the deposition of PDA at dopamine concentrations higher than 0.2 mM and pH values > 3.86, with 5,6-indolequinone as product, which undergoes further oxidation and isomerization processes [19]. Schindler and Bechtold proposed that the anodic oxidation of dopamine in a pH range of 5.8 to 7 involves formation of a semiquinone radical and subsequent deprotonation [18] leading to PDA formation. To date, the final PDA structure has not yet been fully elucidated, similar to the chemical route, although Fourier transform infrared spectroscopy (FTIR) studies indicate poly(5,6-indolequinone) with formation of C-O-C chains during the poly(indole)-like electropolymerization [22]. Single molecule force spectroscopy (SMFS) [23,24] measurements at pulse-deposited PDA have shown polymeric character with contour lengths in the range of 200 nm [17]. For applications, the chemical and physical properties associated with the available functional groups and film morphology play an essential role. To date, no comprehensive study on the influence of the electrochemical deposition method on parameters on electrochemical and nanomechanical properties, such as adhesion, crosslinking, and contour length has been reported. The surface roughness and film thickness of electrodeposited PDA films have been investigated via atomic force microscopy (AFM) [13,16,25,26]. CV, electrochemical impedance spectroscopy (EIS) [27], electrochemical quartz microbalance (EQCM) [19,25], and scanning electrochemical microscopy (SECM) [16,28] have been used to study the electrochemical properties such as charge transfer resistances, ET kinetic, and, associated with this, the permeability of PDA films [6]. The nanomechanical and electrochemical properties of PDA are strongly dependent on the redox state and the pH of the surrounding solution. For example, Ball and coworkers [29] investigated the permeability of differently charged redox species in respect to the deposition method.

In this study, we present a comprehensive investigation of nanomechanical and electrochemical properties of electropolymerized PDA films in dependence of the electrochemical deposition method, namely CV and pulsed deposition. Scanning probe microscopy studies and bulk electrochemical characterization are employed to characterize electrodeposited PDA. In particular, the tunability of adhesion forces based on applied bias or pH, and its dependence on the deposition method, is investigated, as these parameters are important factors in the numerous applications of PDA.

## 2. Materials and Methods

### 2.1. Reagents and Materials

Dopamine hydrochloride was obtained from Sigma-Aldrich (Steinheim, Germany). Sodium hydrogen phosphate (Na_2_HPO_4_), sodium dihydrogen phosphate (NaH_2_PO_4_), potassium chloride (KCl) and hexaammineruthenium (III) chloride ([Ru(NH_3_)_6_]Cl_3_) were purchased from Merck, (Darmstadt, Germany). All solutions were prepared with high purity water (18.1 MΩ·cm, Barnstead Nanopure—Thermo Fisher Scientific, Dubuque, IA, USA). Gold electrodes as working electrodes were obtained via sputter process on silicon. Prior to use, the Au substrates were cleaned in acetone, isopropanol, and highly purified water using an ultrasonic bath. Pt ultra-microelectrodes (UME) (12.5 µm radius and a RG value of 10) were produced by melting the Pt microwire (Goodfellow, Bad Nauheim, Germany) into borosilicate glass (glass capillaries were purchased from Hilgenberg, Malsfeld, Germany) and disc-shaped microelectrodes were exposed via grinding and polishing on diamond lapping films (Allied High-Tech Products, Rancho Dominguez, CA, USA) and on red Technotron cloth (LECO, St. Joseph, MO, USA) using aluminum oxide suspensions (LECO, St. Joseph, MO, USA) as previously described elsewhere [30]. The electrochemical characterization of the UMEs was performed in a three-electrode setup, with an Ag/AgCl/3 M reference electrode (RE), a Pt counter electrode (CE) and the UME as working electrode in a 5 mM hexaammineruthenium (III) chloride/0.1 M KCl solution.

### 2.2. PDA Deposition

PDA depositions were performed using a CHI842B potentiostat (CH Instruments, Austin, TX, USA). For the deposition, a 1 mg/mL dopamine solution in 0.1 M phosphate buffer saline (PBS) (pH 7.0), purged for at least 15 min with Argon to remove dissolved oxygen, was used. Depositions via CV were performed in a potential range of −500 mV to +500 mV vs. Ag/AgCl/3M reference electrode at a scan rate of 10 mV/s with various cycle numbers (5, 10, 15, and 25). Pulsed deposition of PDA was obtained by following pulse cycles: +500 mV/2 s; 0 mV/2 s; −300 mV/2 s; 0 mV/3 s vs. Ag/AgCl/3 M reference electrode with varying pulse cycle numbers (25, 50, 75, 100, and 150 pulse cycles).

### 2.3. SECM Approach Curves

A home-built SECM setup (software: G. Wittstock, University Oldenburg, Oldenburg, Germany) was used to determine ET rates at PDA films deposited with different numbers of cycles or pulse cycles, respectively. Kappa (*ĸ*) values were determined from experimental SECM approach curves (in 5 mM hexaammineruthenium (III) chloride/0.1 M KCl solution) and fittings to theoretical curves following approximations of Cornut and Lefrou [31] using the Mira software (G. Wittstock, University Oldenburg, Oldenburg, Germany) at different substrate potentials (−100 mV, +500 mV vs. Ag/AgCl quasi reference electrode (QRE)) and at different pH values (3, 10). Approach curves are plotted as normalized current *I = I_T_/I_∞_* vs. normalized distance *L = d/r*, where *I_T_* is the recorded tip current in dependence of the distance *d*, and *I_∞_* is the steady state current when the tip is far away from the surface.

### 2.4. AFM Measurements

A 5500 AFM/SPM microscope was used for contact mode AFM measurements (Keysight Technologies, Santa Rosa, AZ, USA) using silicon nitride probes (ORC-8, Bruker AFM probes, CA, USA). Silicon nitride probes were also used for force spectroscopy measurements using a closed loop scanner with 400 repetitive measurements at three different spots at the samples (AFM images were recorded at an area of 50 × 50 μm). The force constant of the cantilevers (nominal *k* = 0.15 N/m) was determined using the thermal noise method [32]. The deflection sensitivity was obtained from the linear slope of the repulsive contact part of the force-displacement curve recorded on a hard substrate. Force spectroscopy measurements were obtained in 0.1 M KCl solution with the PDA films either negatively biased at a potential of −300 mV vs. Ag/AgCl (QRE) or positively biased at a potential of +500 mV vs. Ag/AgCl (QRE). Additionally, force–distance curves were recorded in 0.1 M KCl solutions at pH 3 and pH 10, without applying a bias. All force–distance curves were recorded at a slow sweep rate of 1.0 μm·s^−1^ to minimize hydrodynamic effects, with a loading force of 200 nN. The measured adhesion forces are reported as the mean values ± standard deviation. Statistical analyses are based on Student’s t-test assuming unequal variance. PicoImage (Keysight Technologies, AZ, USA) was used to determine the film thickness and roughness of the PDA films.

### 2.5. Scanning Electron Microscopy (SEM)

SEM imaging were performed with Helios Nanolab 600 FIB/SEM (ThermoFisher, Eindhoven, The Netherlands). SE images were acquired at 1 kV and 86 pA using the immersion mode of the microscope.

### 2.6. EIS Measurements

Electrochemical impedance spectroscopy was conducted using a Metrohm Autolab B.V. N series potentiostat (AUTOLAB PGSTAT 204) with a Fra-32 Module using a three-electrode set-up with the Au-coated substrate (electrode area: 0.28 cm^2^) as working electrode and a Pt wire as the counter electrode using 5 mM [Ru(NH_3_)_6_]Cl_3_ in 100 mM Phosphate buffer (PS) at pH 2.9 and in 100 mM carbonate buffer at pH 10.3. A potential of DC bias of −200 mV vs. Ag/AgCl reference electrode was used for the measurements with an amplitude of +10 mV within a frequency range from 0.1 to 10^5^ Hz. The data analysis and fitting were performed using the Nova 2.1 software (Metrohm Autolab B.V., Utrecht, The Netherlands).

### 2.7. EQCM

EQCM measurements were performed using a Metrohm Autolab B.V. N series potentiostat (AUTOLAB PGSTAT 204) and was conducted on a 0.36 cm^2^ gold-coated AT-cut quartz electrode surface (6 MHz) using an Ag/AgCl reference electrode and Au wire as a counter electrode. Depositions were performed as described under Section 2.2.

### 2.8. Strain-Induced Elastic Buckling Instability for Mechanical Measurements (SIEBIMM)

To determine Young’s modulus of the PDA films in dependence of the electrochemical deposition method, the PDA films were lifted from the electrodes using a polyvinyl alcohol (PVA) support layer as previously described [33]. The PDA/PVA films were transferred to a pre-stretched polydimethylsiloxane (PDMS) layer (~20% strain). The PVA was removed by dissolution in water and the samples were dried at 40 °C. Then, the PDMS strain was relaxed, producing the buckling of the PDA films due to compression. The wavelength *λ* of the wrinkles was determined by AFM (Bruker Dimension ICON) with a Si cantilever (70 kHz resonance frequency and a force constant of 2 N/m in tapping mode). An average over 10 periods was recorded.

## 3. Results and Discussion

### 3.1. Deposition Methods

**Figure 1:**
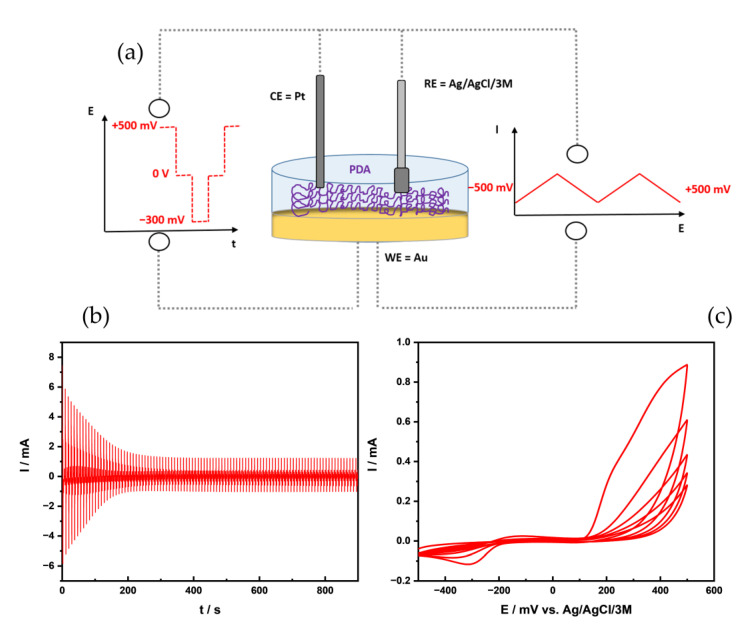
(**a**) Schematic pulse vs. CV PDA deposition at Au macroelectrode. (**b**) I-t curve of pulse deposition (100 pulse cycles) in 1 mg/mL dopamine/0.1 M PBS (pH 7.0) at Au macroelectrode. (**c**) CV of 5 cycles PDA deposition in 1 mg/mL dopamine/0.1 M PBS (pH 7.0) at Au macroelectrode.

As depicted schematically in Figure 1a, PDA films were deposited via voltammetric methods such as CV and pulsed deposition on Au electrodes. The first steps of the electrochemical oxidation of dopamine using CV (Figure 1c) at various pH values are well studied [18,20]; it follows, at higher pH (pH > 7) as used in the presented studies, an ECE mechanism with the formation of dopaminequinone in the first oxidation step at (+500 mV vs. Ag/AgCl). This undergoes cyclization to leucodopaminechrome and further oxidation to dopaminechrome, followed by further oxidation steps leading to the formation of PDA, as shown in Figure S1. The observed decrease in peak currents during consecutive deposition cycles indicates the formation of a blocking layer at the electroactive electrode surface due to the formation of a non-conductive film (Figure 1b,c). For example, for the shown CVs, the peak current at +500 mV vs. Ag/AgCl decreases exponentially by 69.8% after 5 cycles, compared to the current observed at the first cycles.

Less commonly, pulsed deposition is used to obtain electrodeposited PDA films—mainly for layered mixed PDA films [15] or for micro-structured deposition using SECM in direct mode [16]. The formation of PDA using pulsed deposition is shown in Figure 1a,b (100 pulse cycles), respectively. After applying 30 cycles, a drop of 86% could be detected in the faradaic current. Similar to the CV deposition, an exponential decrease over the 100 pulses is observed. It should be noted that a significant contribution of the capacitive current is visible in the current response shown in Figure 1b, which obscures the faradaic contribution for the higher pulse cycle numbers. Pulsed electrochemical deposition has the advantage that the monomer concentration at the electrode surface is essentially renewed after each pulse cycle, as the resting period allows the diffusion of dopamine, which should be fully protonated (given the pH and buffer concentration [18]) towards the electrode surface. The short oxidation pulse then generates a high concentration of reactive species (likely the protonated radical species DPAH_2_^+**.**^), as recently proposed by Schindler and Bechtold [18], that rapidly undergo further reactions, evident by the linearly decreasing current due to film formation.

### 3.2. Film Thickness and Morphology

**Figure 2:**
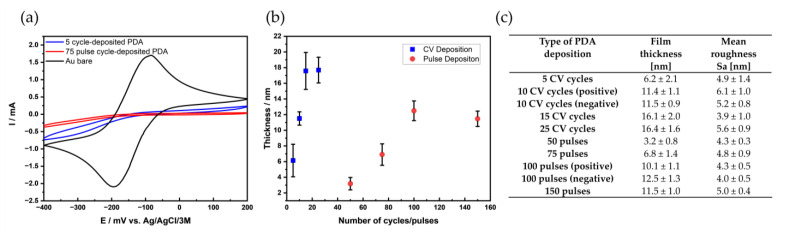
(**a**) CV recorded in 5 mM hexaammineruthenium (III) chloride and 0.1 M KCl before (Au substrate, black) and after PDA deposition (5 CV cycles, red and 75 pulse cycles blue); scan rate 100 mV/s. (**b**,**c**) Thickness of the PDA films based on the number of cycles/pulses. (**c**) Mean roughness (Sa) obtained from AFM images, area: 1 × 1 µm (*n* = 5). Error bars correspond to standard deviation of thickness measurements at 15 different spots.

In the following, the film thickness and film morphology of the films were determined via AFM in dependence of the deposition method, as shown in Figure 2.

Figure 2a shows the CV recorded at a bare Au electrode and at electrodes after modification with PDA. The electrochemical deposition with 5 cycles (CV) results in a film thickness of 6.2 ± 2.1 nm (*n* = 15), which corresponds to a current decrease of 89.4% in comparison to the bare gold electrode. A similar film thickness of 6.8 ± 1.2 nm is obtained via pulsed deposition with 75 pulse cycles (*n* = 15), corresponding to a current decrease of 96.1%. Although the film thickness is similar, the “crosslinking” density of the pulse-deposited films appears to be higher (as shown later through EQCM data) leading to a more effective blocking of the ET (Figure 2a). The film thickness achieved with both deposition techniques was determined via AFM (Figure 2b,c, respectively) and, as expected, with an increasing number of cycles/pulse cycles, the film thickness also increases. The achievable film thickness is strongly dependent on experimental factors such as the concentration of dopamine, pH value and number of deposition cycles, the buffer type and buffer strength concentration, affects the film thickness. Film thicknesses range, depending on the experimental conditions, from several nanometers up to 70 nm [13,16,29]. Deposition with 25 CV cycles resulted in a film thickness of 16.4 ± 1.6 nm and pulsed deposition with 100 pulse cycles resulted in a maximum film thickness of 12.5 ± 1.3 nm. Interestingly, the film thickness obtained for the micro-structured PDA spots using direct mode SECM achieved a value of 75. 4 ± 4.12 nm, applying 90 pulse cycles using the same dopamine concentration but at a lower buffer concentration [16]. Besides the buffer strength, the experimental setup may lead to high deposition rates, as a high concentration of oxidized species is generated in the small gap between the micro-counter electrode and the sample surface leading to enhanced reaction rates and, hence, higher deposition rates. The surface roughness Sa (Figure 2c) of the different films obtained with varying cycles/pulse cycles show a value of about 5 nm with no significant statistically relevant difference in surface roughness values between the different film thicknesses. It should be noted that the surface roughness was determined at areas of 1 × 1 µm avoiding areas with particles shown in Figure S2. It appears that the depositions induced by CV lead to films with a slightly increased number of particles compared to pulse-deposited films, which is also visible in SEM images showing larger areas (Figure S2c,d). Thereby, an increase in the number of larger particles could also be determined for CV-deposited PDA. The determined surface roughness values are comparable with values reported in the literature for films obtained by dip coating [34,35,36,37], and electrodeposited PDA films on Au electrodes [38], as well as micro-structured films [16]. However, for many reported values, the surface roughness was determined at relatively small areas. It should be noted that the formation of the blister-like, morphology pulse-deposited micro-structured film was not observed in the bulk experiments.

### 3.3. SECM Kinetic Studies

Among the unique properties of PDA films is the change of properties such as interactions (adsorption) of proteins and living cells dependent on the redox state of the functional groups of PDA [39]. The redox state of PDA can be studied via the anodic and cathodic peak separation of CV using outer-sphere redox active species at PDA modified electrodes [29]. Recording SECM approach curves also gives information on the electron transfer kinetic, as shown for “electrode fouling” at carbon-based electrodes in dopamine solution [28]. The advantage of this method is that the heterogeneity of a sample in respect to electron transfer is probed. The ET behavior was here evaluated in dependence of the deposition method, the film thickness, and the applied potential or pH value of the solution. The ET is reported as dimensionless rate constant *ĸ* ($ĸ=\frac{k_{\mathrm{eff}}*r}{D}$), where *D* is the diffusion coefficient of the redox mediator, *r* the active radius of the tip, and *k_eff_* the constant of the apparent heterogeneous ET rate [40]. Redox active groups, such as the quinone/hydroquinone couple present in the deposited films, can be reduced, oxidized by applying moderate positive or negative substrate potential, respectively [16,17].

**Figure 3:**
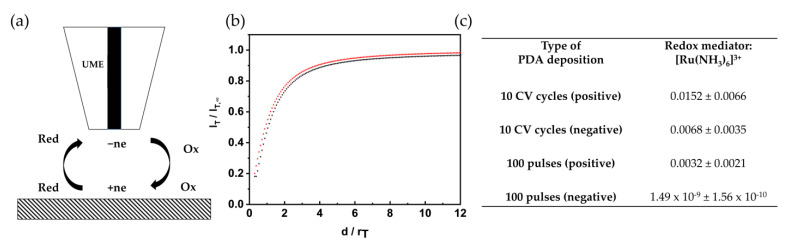
(**a**) Feedback mode of SECM. (**b**) Approach curve recorded in 5 mM hexaammineruthenium (III) chloride/0.1 M KCl with a scan velocity of 1 µm/s; theoretical approach curve were obtained using fitting by Cornut and Lefrou [40] (black); experimental approach curve recorded at 100 pulse cycles deposited PDA film with a positive ending potential (red). (**c**) Dimensionless rate constant *κ* in dependence of ending potential of the depositions (*n* = 15).

Figure 3a shows schematically the principle of feedback mode SECM, along with an exemplary, normalized current vs. normalized distance values (Figure 3b, black curve), recorded for a positively charged redox species, and the theoretical curve (red curve), based on work from Cornut and Lefrou [31]. A positively charged redox mediator was used for all films investigated in this study. Table 1 summarizes the *ĸ* values in relation to the deposition mode and the potential applied to the PDA-modified electrodes after deposition. At a negative sample potential (−100 mV vs. Ag/AgCl (QRE)), the quinone/hydroquinone couple should be in the reduced state. As measurements were performed at a neutral pH value (experiments were performed in 0.1 M KCl) with the given pKa values of the phenolic groups and amino group [18], it is expected that the films are less permeable for the positively charged redox mediator, which is in accordance with the *ĸ* values obtained for the films deposited via CV (5 and 10 cycles). Interestingly, the film deposited via pulse deposition (100 pulses), which has a film thickness similar to the film deposited with 10 cycles, shows a significant decrease in ET, which may be explained with a different film morphology reflecting a more cross-linked film. At a positive potential (+500 mV vs. Ag/AgCl (QRE)), the variations in ET kinetic based on the film thickness and deposition methods are less pronounced, which can be explained by the presence of more quinoid groups and therefore a less pronounced electrostatic effect. The redox state of the PDA is important for applications, e.g., in biomedical research [41], as increased amount of quinone moieties enhance the ability for biomolecule adhesion. Approach curves were also recorded at films deposited with only 25 pulse cycles, as shown in Figure S3, which clearly leads to less blocking films as expected with kappa values for the shown curves: 0.1018 (olive), 0.0615 (magenta), and 0.0507 (cyan). The curves were recorded at different spots of the sample, which may be explained with the reduced film thickness or the formation of a less uniform coating.

The ET kinetic behavior was also investigated in dependence of the surrounding pH without applying potential to the PDA-modified substrate. The zwitterionic nature of PDA due to the amino and phenolic groups and the associated pH switchable permselectivity has been investigated by Yu et al., for films obtained by dip-coating [42]. The authors performed CV and electrochemical impedance spectroscopy studies in pH 3, 7, and 11 using a negatively charged (ferro/ferricyanide couple) and a positively charged (hexaammineruthenium (III) chloride) redox species. At basic pH values, PDA has a net negative charge that allows cations to pass, whereas at low pH values, the film is positively charged. The latter leads to the impediment of the diffusion of the positively charged redox species into the film, but it is more permeable for the negatively charged redox species. In accordance with the observations for films obtained by dip-coating, we report here quantitative values for ET kinetics. At pH 3, electrostatic repulsion—along with reduced *ĸ* values—are observed for all films, independent of the film thickness (5, 10 CV cycles, 100 pulses), for the positively charged redox species, as summarized in Table 1. For studies in basic solutions, pH 10 was chosen, as the strong adhesion properties of PDA at higher pH values may lead to a delamination of the film as reported by Wei for polymer membranes [43]. At pH 10, the phenolic groups and the amino groups are deprotonated, which is reflected by the observed *ĸ* values with a significantly increased electron transfer rate constant. The values are similar for all investigated films, independent of the electrochemical deposition method.

The ET kinetics are also influenced by the ending potential of the deposition sequence, as it determines the final redox state of the film once no further external potential is applied. For the CV-induced deposition, the potential was cycled between −500 and +500 mV vs. Ag/AgCl/3M, and both reverse potentials were chosen as ending potential in the studies presented in Figure 3. For pulse deposition, the ending pulse potential was chosen at −300 mV and +500 mV vs. Ag/AgCl/3M, respectively. Approach curves were again recorded in hexaammineruthenium (III) chloride/KCl solution. Figure 3b represents an approach curve recorded at a PDA film deposited with 100 pulse cycles and ending-potential of +500 mV vs. Ag/AgCl/3M (in comparison to the theoretical curve). A similar trend, as in the case of applied potential, could be observed Figure 3c. The pulse-deposited PDA film (100 pulse cycle) stopped at negative potential shows a significantly lower *ĸ* value compared to the CV-deposited films, and the pulse-deposited film stopped at positive potential, which is in good agreement with the data obtained for biased samples. The electrochemical properties of the deposited PDA films can already be controlled by the ending potential during electrochemical deposition.

### 3.4. Electrochemical Impedance Spectroscopy (EIS) Studies

Electrochemical impedance spectroscopy measurements at different pH values were evaluated to collect more detailed insight in the pH dependence of the ET behavior. EIS has been used by several researchers to characterize PDA or analogue films under different experimental conditions [25,42,44,45]. The charge transfer resistance (*R_ct_*) at the electro-deposited PDA films were quantitatively evaluated from the diameter of the obtained semicircle of the Nyquist plots.

**Figure 4:**
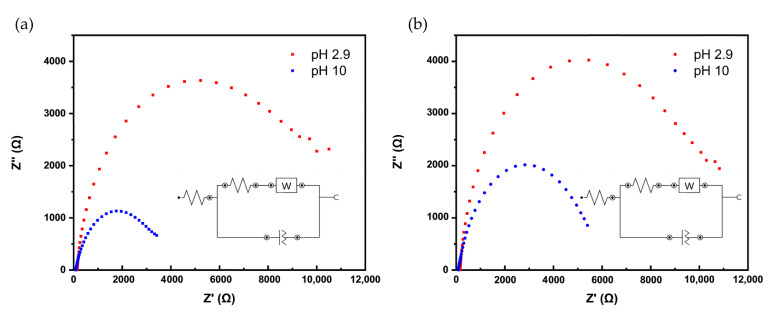
Nyquist plots at two different pH values (pH 2.9 and pH 10). Impedance spectra were recorded with a bias potential of −200 mV applied to the sample with an amplitude of 10 mV. Spectra were recorded in a frequency range of 0.1 to 10^5^ Hz in hexaammineruthenium (III) chloride as redox probe. (**a**) PDA deposited with 10 CV cycles. (**b**) PDA deposited with 100 pulse cycles.

The recorded EIS spectra, using hexaammineruthenium (III) chloride as redox probe, are shown in Figure 4a,b, respectively. The Nyquist plots reflect the data recorded at acidic and basic pH values (2.9 or 10) at the electrodeposited films (10 cycles and 100 pulse cycles, respectively). The data were modelled using a Randles circuit with a constant phase element (CPE), as shown in the insets of Figure 4. In the chosen frequency range between 100 kHz and 0.1 kHz, the plots indicate that the resistance is mainly driven by heterogeneous ET, indicating that the electrode reaction at the film interface is a kinetically controlled process.

The extracted *R_CT_* values for both pH values in dependence of the electrochemical deposition method are listed in Table 2. As expected, for pH 2.9 with protonated amino groups of the PDA film, the charge transfer resistance is 50% higher than pH 10 for the positively charged redox species. A similar trend based on pH was reported for the same positively charged redox species by Yu et al. [42] for CV-deposited films, although the experimental parameters for deposition of PDA and the EIS experiments were different. There is no significant difference of charge transfer resistance for the two deposition methods, which is expected, as the film thickness is similar (see Figure 1c). However, it should be noted that standard deviation for replicate measurements were significantly higher for the pulse-deposited films.

### 3.5. Electrochemical Quartz Microbalance (EQCM)

**Figure 5:**
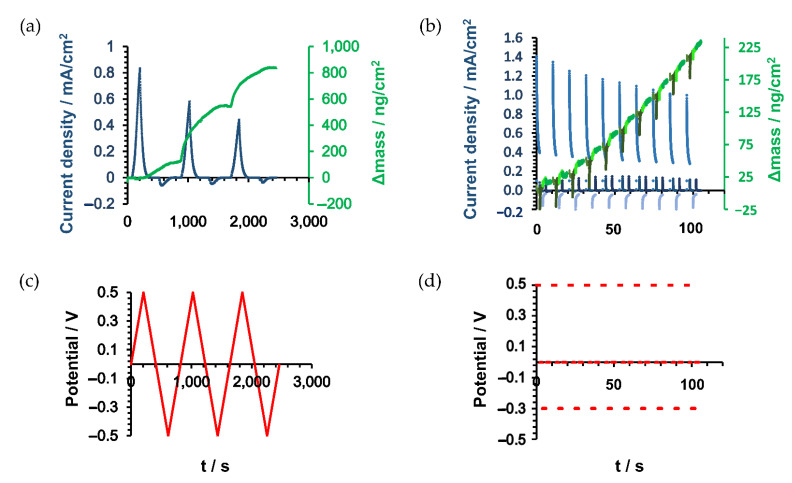
(**a**) Current density (blue) and areal mass density (green) for 3 CV cycles. (**b**) Current density (blue) and areal mass density (green) for 10 pulse cycles. (**c**) E-t curve for the first 3 CV cycles. (**d**) E-t curve for the first 10 pulse cycles.

Differences in the mass of PDA during the electropolymerization were assessed by EQCM. EQCM has been used by Li et al., to investigate the oxidation of dopamine in respect to DA concentration, solution pH, the electrolyte salt, and potential-sweep rate using EQCM [19]. The first 3 CV cycles and first 10 pulse cycles are presented in Figure 5. During CV deposition, the current for the oxidation of catechol groups to quinone peaks around +300 mV, and subsequently reduces as the applied potential is reversed. The current density (Figure 5a) steadily decreases with each CV cycle due to the deposition of the PDA film. In respect to deposited mass, the first cycle only corresponds to a small mass change, but the deposition rate jumps abruptly as the applied potential approaches +500 mV with a deposition of 119.6 ng/cm^2^; the second CV resulted in a deposition of 433.4 ng/cm^2^ and then maintains a linear deposition rate of around 220 ng/cm^2^ until the 7th CV. The observable slight depression in mass deposition that is observed in the massograme may be associated to oxidized unbound intermediates diffusing from the surface, before again rapidly increasing as the applied potential approaches +500 mV.

This behavior can be observed continually over 10 cycles (Figure S4a) indicating a well-controlled deposition of PDA with a total mass change of deposited PDA of 2500 ng/cm^2^ for 10 cycles. A slight decrease in mass deposition is observed for the last three cycles. The pulsed deposition exhibits a more complex response in current, as well as in mass change. The initial pulse at +500 mV generates an oxidation current that is higher in comparison to the CV induced deposition. The peak current also reduces with each pulse cycle, but at a lower rate. The mass deposition per cycle increases upon 16 pulse cycles with a maximum deposition of 32.4 ng/cm^2^ per cycle. After this, the deposition rate slows asymptotically over the remainder of the 100 cycles, which indicates that the ET is more effectively hindered (Figure S4b). As a consequence, the final mass deposition for the 100 pulse cycles is with 1480 ng/cm^2^ significantly lower than for 10 CV cycles. Despite a similar film thickness (see Figure 1c) and significantly lower area density, it appears that pulse-deposited PDA films show stronger cross-linking, which is in accordance with the obtained kappa values.

### 3.6. AFM Force Spectroscopy

**Figure 6:**
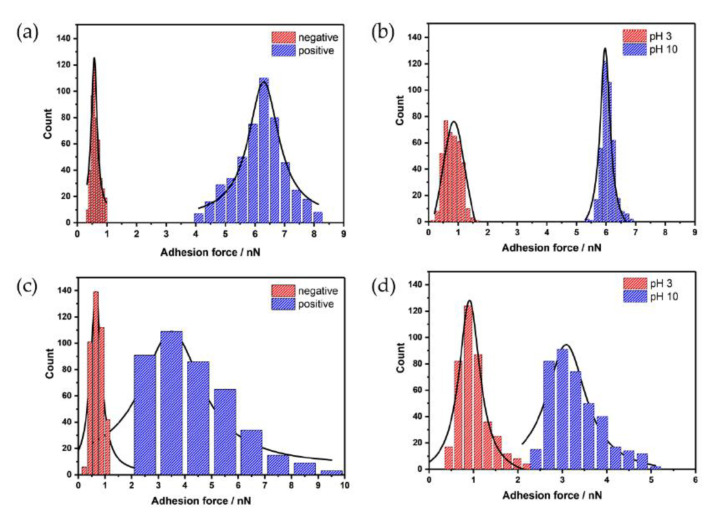
Histograms of adhesion forces. (**a**,**b**) pulse-deposited PDA film (100 pulse cycles); (**a**) at different applied potentials (−300 mV vs. Ag/AgCl (red) and +500 mV vs. Ag/AgCl (blue)); (**b**) PDA film immersed in solutions with different pH values (pH 3, red and pH 10, blue); (**c**,**d**) PDA film deposited via CV (10 cycles); (**c**) at different applied potentials (−300 mV vs. Ag/AgCl (red) and +500 mV vs. Ag/AgCl (blue)); and (**d**) PDA film immersed in solutions with different pH values (pH 3, red and pH 10, blue); (*n* = 400).

PDA films exhibit excellent adhesion properties [5,9,46,47], which can be quantitatively determined via AFM force spectroscopy. Up to now, force spectroscopy studies on PDA films have been predominantly used for films obtained via the chemical deposition route, e.g., for the determination of the contour length of polymeric chains [12]. Additionally, the influence of thermal annealing on the adhesion properties [48] and the influence of wettability of SAM-modified gold electrodes on adhesion properties of PDA has also been investigated via force spectroscopy [34]. Here, we investigate differences in adhesion properties of electro-deposited PDA films in dependence of the electrochemical deposition method, the applied potential after deposition, and the influence of the pH value. All force curves were recorded in a solution with relatively high ionic strength (0.1 M KCl) to screen electrostatic interactions. Figure 6 shows histograms of the mapped adhesions forces under the different experimental conditions. Again, PDA films with similar film thickness were investigated (10 CV cycles and 100 pulse cycles). As previously observed with pulse-deposited PDA-coated colloidal AFM probes [17], based on the applied potential, strong adhesion forces with an average adhesion force of 6.20 ± 0.83 nN (*n* = 400) were observed between the oxidized surface groups (quinoid groups) of the pulse-deposited film and a hydrophilic AFM tip. Interestingly, for CV-deposited films, the difference in measured adhesion for positively biased PDA film is less pronounced, with an average adhesion force of 3.87 ± 1.59 nN. For negatively biased films, independent of the electrochemical deposition mode, the observed adhesion forces are 0.63 ± 0.18 nN (pulse-deposited) and 0.59 ± 0.21 nN (CV-deposited). The observed differences at applied positive potential may indicate differences in film morphology, which is also evident by the broader distribution of the adhesion forces for the CV-deposited films. The applied potential has a stronger influence on the distribution at positive bias for both deposition methods, which may be associated with potential-induced conformational changes as already reported for pulse deposition of PDA on conductive colloidal probes [17]. Analogously, a strong dependence of adhesion forces was also observed at different pH values. At pH 3, the functional groups (e.g., amino groups) of PDA are protonated, resulting in reduced adhesion with the silicon nitride AFM probe, which is dominated by positive charges at pH 3 [49]. At pH 10, the measured adhesion forces are similar to the values obtained for the oxidized films, which may be explained by the fact that the pH studies were undertaken at the films, which were biased prior to the pH study at positive potentials. It should be noted that, for pH 10, a more narrow distribution of adhesion forces was obtained, independent of the deposition method, whereas for pH 3, the distribution in adhesion forces was slightly higher. For CV-deposited films, the broader adhesion force distribution may be related to the fact that, qualitatively, the films seem to show more particles (Figure S2) at the surface known from dip coated films.

Single molecule force spectroscopic measurements at polymers also provide access to information on the degree of crosslinking of the polymer, i.e., via the contour length of polymer chains and rupture forces. Thereby, information on molecular assemblies and the inter- and intramolecular forces, such as electrostatic, hydrogen bonding, π–π stacking, etc., is obtained [50]. In respect to the measured contour length, plateaus, and rupture forces, we could observe differences between the deposition methods as exemplarily shown in Figure S5. PDA films deposited with CV revealed that 16% of the 1600 evaluated force curves showed contour lengths in the range of 80 to 900 nm for the different experimental conditions (applied bias and pH of solution), whereas the highest contour lengths were observed for positively charged films (average 560 nm ± 252 nm). For pulse-deposited films, only 8% of the evaluated force curves (1600) showed contour lengths ranging from 80 to 700 nm, with the highest contour lengths for films immersed in pH 10 (average 356 nm ± 136 nm). In comparison to experiments described in the literature [12,17,48] on films obtained by dip-coating and films electrodeposited from buffered solution with lower ionic strength, we observe for both electrodeposition methods a higher percentage of curves showing the pulling of polymer chains, most exhibiting several plateaus with a plateau length pattern as shown in Figure S5. Such plateau patterns are characteristic for peeling events, observed for polymers with weak interaction to the substrate [51]. Pulse-deposited films exhibit stronger intermolecular interaction between polymeric chains, likely due to an increased degree of cross-linking, which can be derived from the reduced pulling events. This is also in agreement with the electrochemical data presented here. Some of the recorded force curves also showed rupture features (spikes), as previously described by Messerschmidt and co-workers as single molecule stretching events [12].

### 3.7. SIEBIMM

The nanometer thickness of the electro-deposited PDA films makes it difficult to determine their mechanical properties using conventional techniques such as universal testing machines or nanoindentation [52]. Hence, the nanomechanical properties of the deposited films were characterized via strain-induced elastic buckling instability for mechanical measurements (SIEBIMM), which was recently developed to determine the elastic moduli of thin polymeric films [53]. Compression of a bilayer of the film of interest on a soft, thick substrate such as PDMS induces wrinkling of the film. The wavelength of the wrinkles is dependent on the thickness of the film and the difference in Young’s modulus of the film and PDMS, allowing quick determination of the Young’s modulus of thin films. Using a technique we previously described [33], the PDA films were transferred to pre-stretched PDMS slabs. Upon relaxation of the strain, wrinkling of PDA films was determined. The wavelength, λ, of the wrinkles was measured by AFM, as shown in Figure S7, and the Young’s modulus was calculated using the following equation:

(1) $$E_{n}=\frac{3\left(E_{s}\left(1-\nu_{n}^{2}\right)\right)}{\left(1-\nu_{s}^{2}\right)}{\left(\frac{\lambda }{2\pi t}\right)}^{3}$$

**Figure 7:**
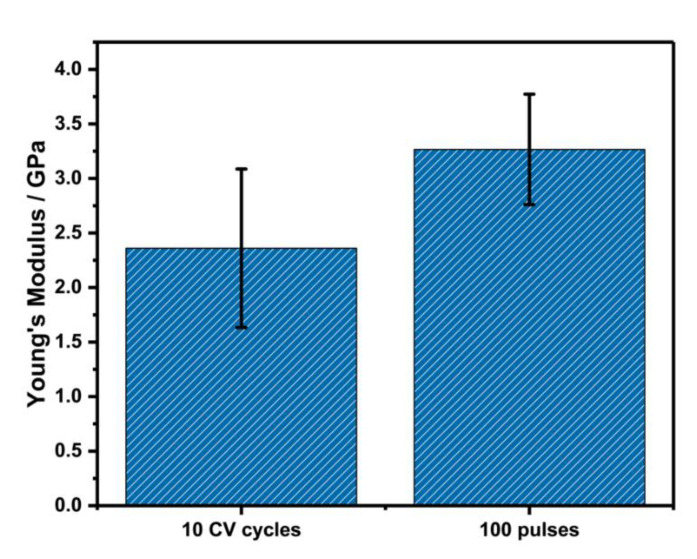
Histograms of Young’s modulus of CV- and pulse-deposited PDA films (10 CVs; 100 pulse cycles); (*n* = 3 reflecting three different samples).

*E* is the Young’s modulus, *t* is the thickness of the PDA films, ν is the Poisson’s ratio, and the subscripts *n* and *s* refer to PDA film and PDMS, respectively. A value of 2.50 MPa was used for the Young’s modulus (*E_S_*) and 0.50 and 0.33 were taken for the Poisson’s ratios of the PDMS and PDA films, respectively [54]. The Young’s modulus for the pulse-deposited film was found to be slightly higher than for the CV-deposited film (Figure 7). The increased modulus of the pulse-deposited film again points to the fact that pulse deposition results in a higher degree of crosslinking or fewer internal defects compared to the CV-deposited film, which is also in agreement with the obtained electrochemical data.

A Young’s modulus for the pulsed film of 3.27 ± 0.51 GPa was determined which is slightly higher than for the CV film 2.36 ± 0.73 GPa. Similar values have already been shown for dip-coated PDA films [54]. Nonetheless, the modulus of both films was within the range of other polymeric films measured using the SIEBIMM method [53,55,56].

## 4. Conclusions

In this study, the properties of electrodeposited thin PDA films, obtained via CV and pulse deposition, have been investigated in respect to their electrochemical and nanomechanical properties, which play a significant role in most applications. Scanning probe microscopy and bulk electrochemical methods were used in these studies to compare the physical and electrochemical properties. It was revealed that there is a significant difference in ET kinetics based on the applied potential and pH value, if the film is immersed after deposition. Increased ET kinetics were determined for the positively biased film, which contains mainly oxidized functional groups (quinone). In addition, SECM and EQCM studies revealed that pulse-deposited PDA films appear to be increasingly cross-linked, as evidenced by limited ET in comparison to CV-deposited films. One of the unique features of PDA are the pronounced adhesion forces. AFM force spectroscopic measurements of pulse-deposited and CV-deposited show a distinct difference in adhesion forces based on the applied potential and pH of the solution. The polymeric contribution of the obtained films was also evaluated by determining the contour length and plateaus of the retract force curves. It is clearly evident that the percentage of force curves showing such polymer chain pulling events is much higher compared to films obtained by dip coating. In total, 16% of force curves for the CV-deposited films revealed contour lengths with highest values up to 900 nm. Using SIEBIMM, an increased Young’s modulus could be detected for pulse-deposited PDA films compared to CV-deposited PDA films. This supports the hypothesis derived from force spectroscopy that pulse-deposited PDA films are characterized by a more dense and cross-linked structure compared to CV-deposited PDA films, which is in line with the obtained electrochemical data.

## Figures and Tables

**Table 1:** Dimensionless rate constant *κ* in dependence of substrate potential and pH value (*n* = 15 for 5 cycles, *n* = 12 for 10 cycles, and *n* = 15 for 100 pulse cycles, respectively).

Type of PDA Deposition	Substrate Potential vs. Ag/AgCl: Redox Mediator: [Ru(NH_3_)_6_]^3+^		Redox Mediator: [Ru(NH_3_)_6_]^3+^ in Different pH	
	−100 mV	+500 mV	pH 3	pH 10
**5 cycles**	0.0027 ± 0.0015	0.0139 ± 0.0078	2.95 × 10^−10^ ± 5.67 × 10^−10^	0.0074 ± 0.0041
**10 cycles**	0.0025 ± 0.0014	0.0090 ± 0.0018	4.37 × 10^−10^ ± 1.15 × 10^−10^	0.0075 ± 0.0025
**100 pulse cycles**	1.44 × 10^−9^ ± 4.03 × 10^−10^	0.0162 ± 0.0121	1.22 × 10^−9^ ± 1.01 × 10^−10^	0.0040 ± 0.0036

**Table 2:** *R_ct_* calculated from the fitting Randles circuit model of the impedance data using hexaammineruthenium (III) chloride as redox probe (each value was determined at two freshly prepared depositions).

pH	*R_ct_* (kΩ) [Ru(NH_3_)_6_]^3+^	
	10 CV cycles	100 pulse cycles
2.9	7.3 ± 1.1	7.6 ± 1.6
10.0	3.7 ± 0.45	3.6 ± 2.0

## Data Availability

The data presented in this study are available in the manuscript and the Supplementary Materials.

## Supplementary Materials

The following are available online at https://www.mdpi.com/article/10.3390/nano11081964/s1, Figure S1: First steps of electrochemical oxidation of dopamine in slight basic solution. A ECE mechanism is described in the literature. In the first electrochemical step dopamine quinone is formed, which undergoes a cyclization reaction (chemical step), which furthers oxidizes dopaminechrome and 5,6-dihydroyindole, respectively. Figure S2: Comparison of AFM contact mode images (recorded in air) and SEM images using CV and pulsed deposition (topography). (a) AFM image: PDA deposited with 5 cycles (CV), (b) AFM image: PDA depsoited with 75 pulse cycles (c) SEM image of PDA deposited with 10 cycles (CV), (d) PDA deposited with 100 pulse cycles. Figure S3: SECM approach curves recorded at three different spots on 25 pulse-deposited unbiased PDA substrate in 5 mM hexaammineruthenium (III) chloride and 0.1 M KCl. Determined *ĸ* values: 0.1018 (olive), 0.0615 (magenta) and 0.0507 (cyan). Simulated approach curve for negative feedback (red) and positive feedback (green) in relation to RG 10. Figure S4: (a) Current density (blue) and areal mass density (green) for 10 cycle-deposited PDA film. (b) Current density (blue) and areal mass density (green) for 100 pulse-deposited PDA film. Figure S5: Exemplary force-distance curve recorded in 0.1 M KCl by applying a substrate potential of (a) −300 mV and (b) +500 mV vs. Ag/AgCl (QRE) at a 100 pulse-deposited PDA-modified Au substrate, (c) −300 mV and (d) +500 mV vs. Ag/AgCl (QRE) on 10 cycle-deposited PDA-modified Au substrate. Figure S6: Distribution of plateau lengths of the force-distance curves at different PDA-modified Au substrate. (a) 10 cycle-deposited PDA (b) 100 pulse-deposited PDA. Figure S7: AFM topography images of wrinkled PDA films on PDMS. (a) PDA film deposited with 10 CV cycles, (c) line profile retracted from (a) as marked with line. (b) PDA film deposited via 100 pulse cycles, (d) line profile retracted from (b) as marked with line.
