# Supplementary material for: Physicochemical and Electrochemical Characterization of Electropolymerized Polydopamine Films: Influence of the Deposition Process
Source: Nanomaterials (Basel). 2021 Jul 30;11(8):1964. doi: 10.3390/nano11081964 (PMC8400158; doi:10.3390/nano11081964)
Supplement: Supplementary file 1 [file nanomaterials-11-01964-s001.zip › nanomaterials-1285925-supplementary.pdf]

Supplementary Materials

Physicochemical and Electrochemical Characterization of Electropolymerized Polydopamine Films: Influence of the Deposition Process

Julian Kund [1], Sven Daboss [1], Tommaso Marchesi D'Alvise [2], Sean Harvey [2], Christopher V. Synatschke [2], Tanja Weil [2] and Christine Kranz [1,*]

[1] Institute of Analytical and Bioanalytical Chemistry, Ulm University, Albert-Einstein-Allee 11, 89091 Ulm, Germany; julian.kund@uni-ulm.de (J.K.); sven.daboss@uni-ulm.de (S.D.)
[2] Max Planck Institute for Polymer Research, Ackermannweg 10, 55128 Mainz, Germany; marchesi@mpip-mainz.mpg.de (T.M.D.); sdharv@gmail.com (S.H.); synatschke@mpip-mainz.mpg.de (C.V.S.) weil@mpip-mainz.mpg.de (T.W.)
* Correspondence: christine.kranz@uni-ulm.de; Tel.: +49-731-50-22749

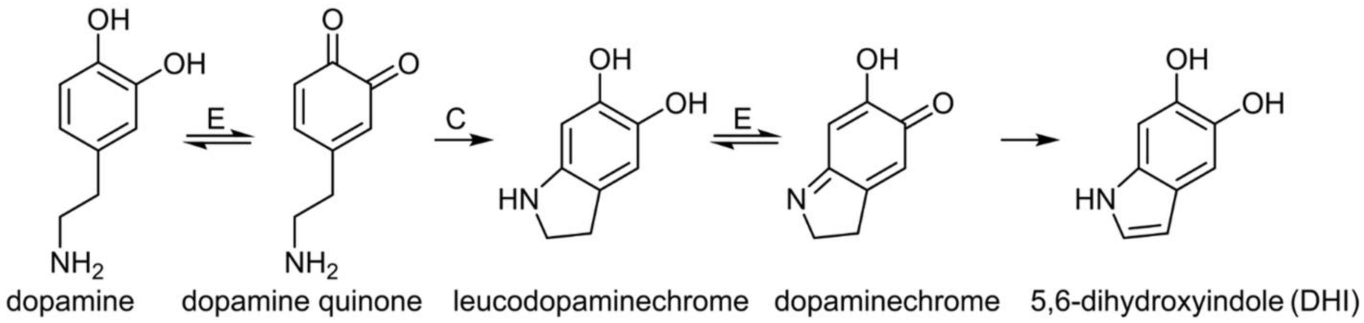

Figure S1. First steps of electrochemical oxidation of dopamine in slight basic solution. A ECE mechanism is described in the literature. In the first electrochemical step dopamine quinone is formed, which undergoes a cyclization reaction(chemical step), which furthers oxidizes dopaminechrome and 5,6-dihydroyindole, respectively.

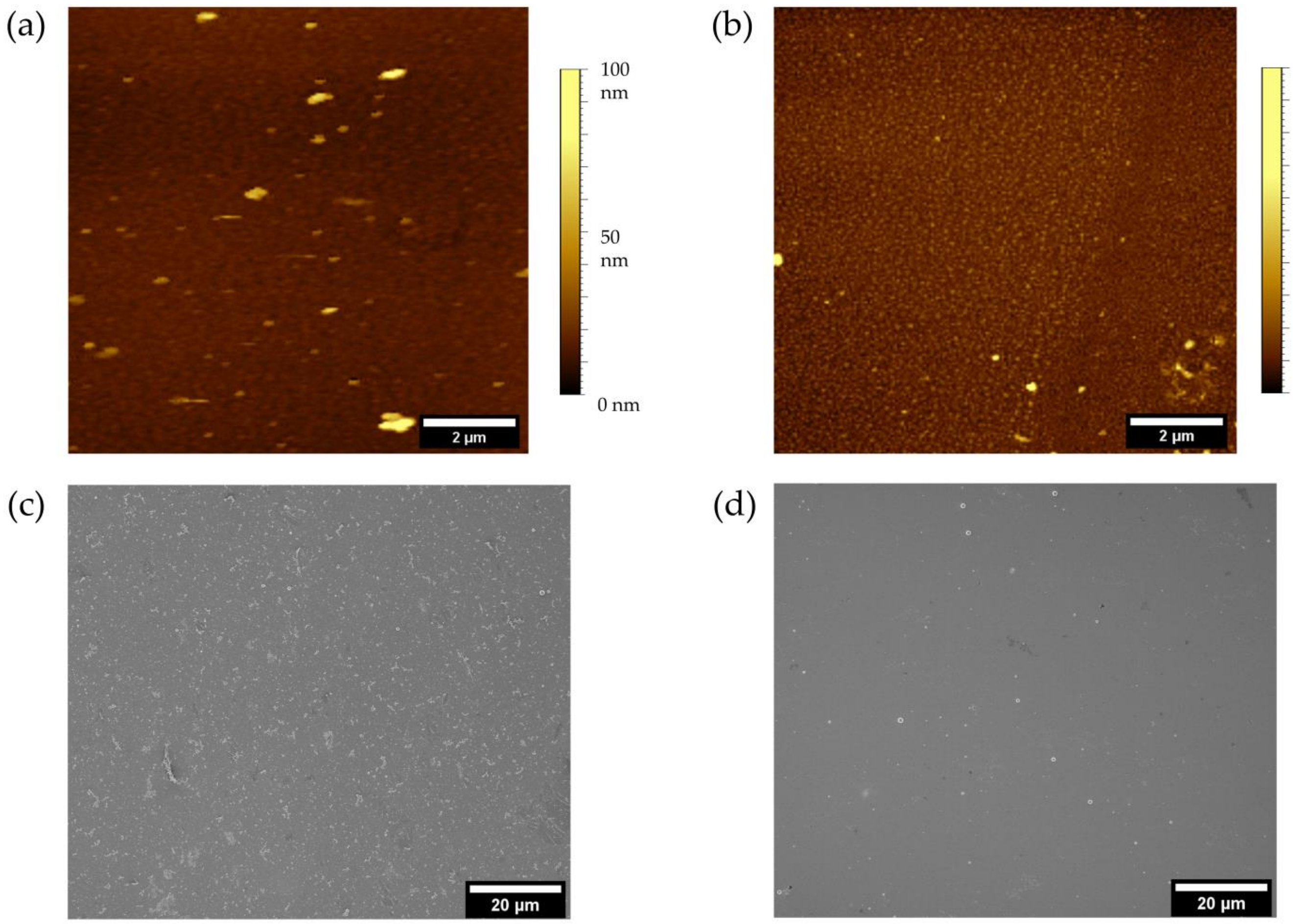

Figure S2. Comparison of AFM contact mode images (recorded in air) and SEM images using CV and pulsed deposition (topography). (**a**) AFM image: PDA deposited with 5 cycles (CV), (**b**) AFM image: PDA depsoited with 75 pulse cycles (**c**) SEM image of PDA deposited with 10 cycles (CV), (**d**) PDA deposited with 100 pulse cycles.

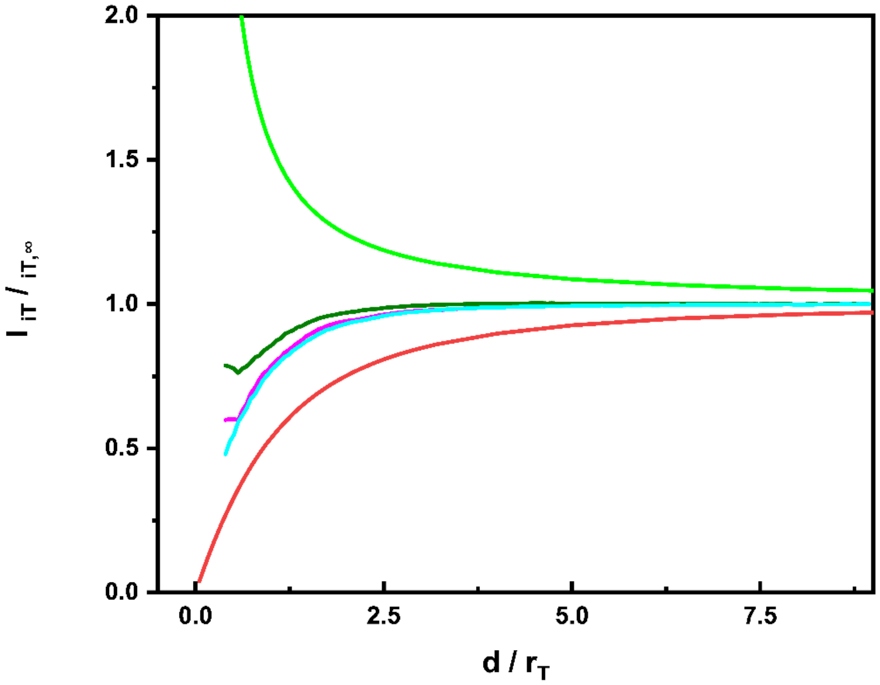

Figure S3. SECM approach curves recorded at three different spots on 25 pulse-deposited unbiased PDA substrate in 5 mM hexaammineruthenium (III) chloride and 0.1 M KCl. Determined κ values: 0.1018 (olive), 0.0615 (magenta) and 0.0507 (cyan). Simulated approach curve for negative feedback (red) and positive feedback (green) in relation to RG 10.

(a) (b)

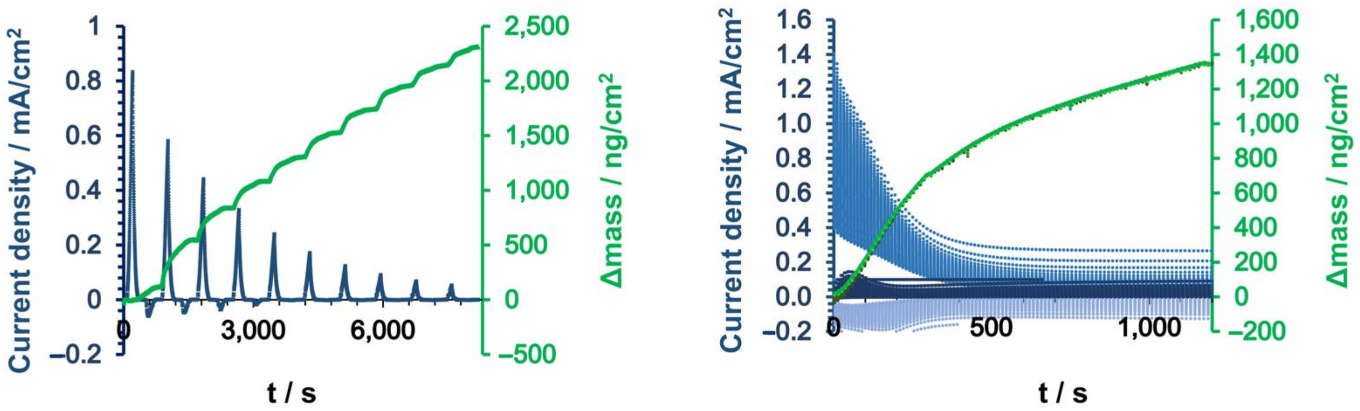

Figure S4. (**a**) Current density (blue) and areal mass density (green) for 10 cycle-deposited PDA film. (**b**) Current density (blue) and areal mass density (green) for 100 pulse-deposited PDA film.

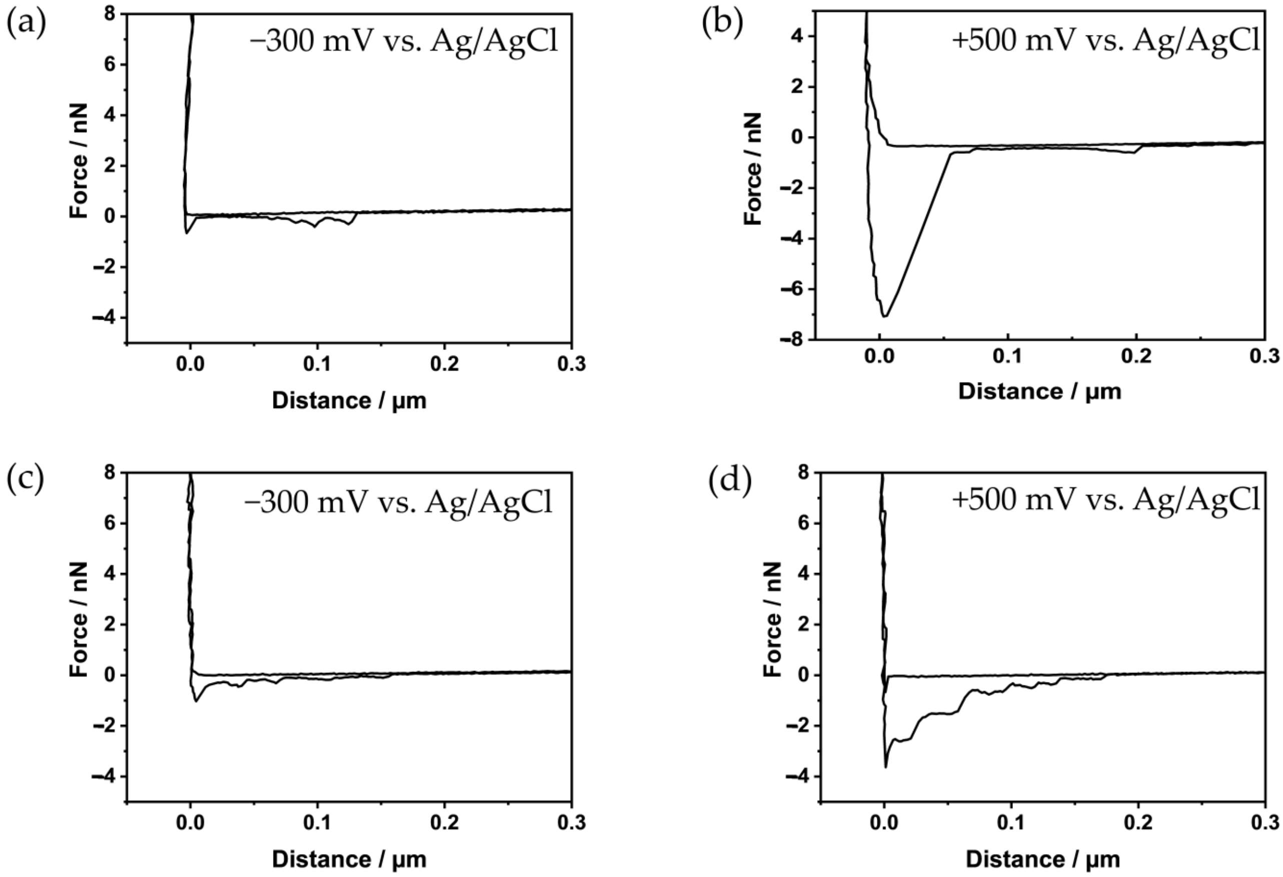

Figure S5. Exemplary force-distance curve recorded in 0.1 M KCl by applying a substrate potential of (**a**) −300 mV and (**b**) +500 mV vs. Ag/AgCl (QRE) at a 100 pulse-deposited PDA-modified Au substrate, (**c**) −300 mV and (**d**) +500 mV vs. Ag/AgCl (QRE) on 10 cycle-deposited PDA-modified Au substrate.

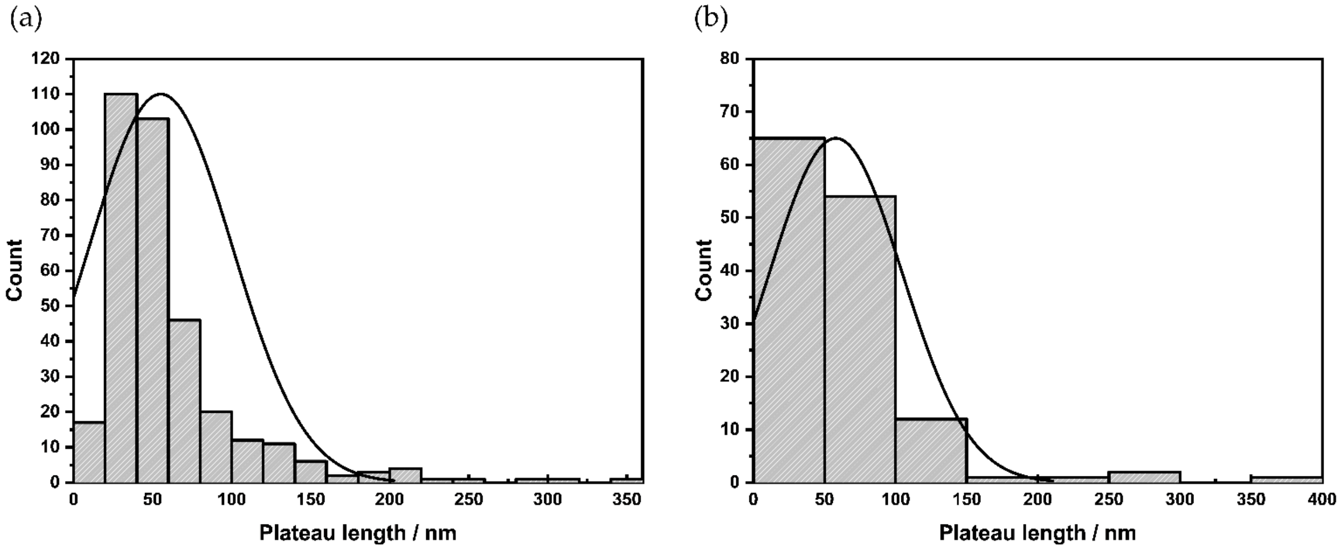

Figure S6. Distribution of plateau lengths of the force-distance curves at different PDA-modified Au substrate. (**a**) 10 cycle-deposited PDA (**b**) 100 pulse-deposited PDA.

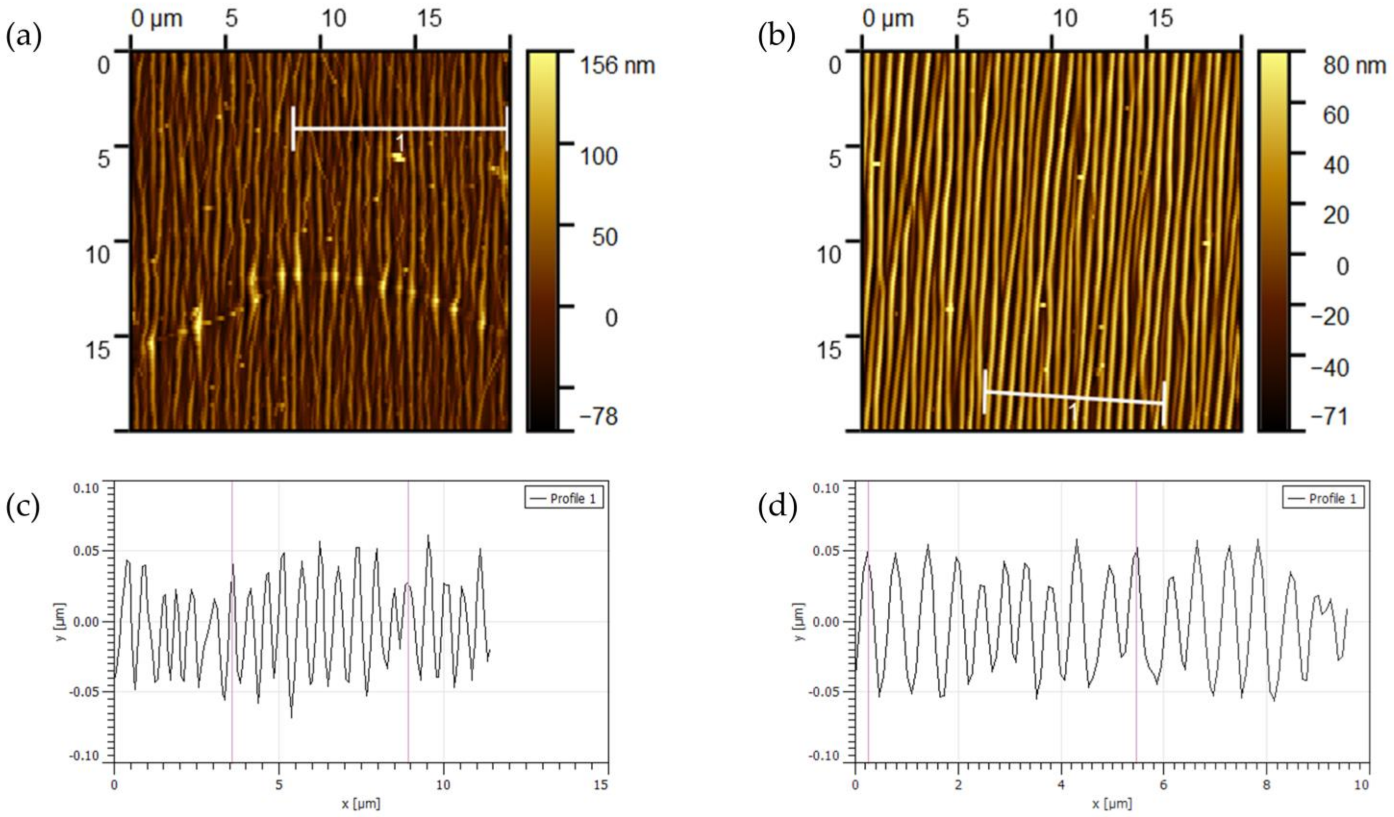

Figure S7. AFM topography images of wrinkled PDA films on PDMS. a) PDA film deposited with 10 CV cycles, (**c**) line profile retracted from a) as marked with line. (**b**) PDA film deposited via 100 pulse cycles, (**d**) line profile retracted from b) as marked with line.
